# A Cross-Domain Weakly Supervised Diabetic Retinopathy Lesion Identification Method Based on Multiple Instance Learning and Domain Adaptation

**DOI:** 10.3390/bioengineering10091100

**Published:** 2023-09-20

**Authors:** Renyu Li, Yunchao Gu, Xinliang Wang, Junjun Pan

**Affiliations:** 1 State Key Laboratory of Virtual Reality Technology and Systems, Beihang University, Beijing 100191, China; zy2106319@buaa.edu.cn (R.L.); wangxinliang@buaa.edu.cn (X.W.); pan_junjun@buaa.edu.cn (J.P.); 2 Hangzhou Innovation Institute, Beihang University, Hangzhou 310051, China; 3 Research Unit of Virtual Body and Virtual Surgery Technologies, Chinese Academy of Medical Sciences, 2019RU004, Beijing 100191, China

**Keywords:** diabetic retinopathy identification, multiple instance learning, weakly supervised learning, cross-domain

## Abstract

Accurate identification of lesions and their use across different medical institutions are the foundation and key to the clinical application of automatic diabetic retinopathy (DR) detection. Existing detection or segmentation methods can achieve acceptable results in DR lesion identification, but they strongly rely on a large number of fine-grained annotations that are not easily accessible and suffer severe performance degradation in the cross-domain application. In this paper, we propose a cross-domain weakly supervised DR lesion identification method using only easily accessible coarse-grained lesion attribute labels. We first propose the novel lesion-patch multiple instance learning method (LpMIL), which leverages the lesion attribute label for patch-level supervision to complete weakly supervised lesion identification. Then, we design a semantic constraint adaptation method (LpSCA) that improves the lesion identification performance of our model in different domains with semantic constraint loss. Finally, we perform secondary annotation on the open-source dataset EyePACS, to obtain the largest fine-grained annotated dataset EyePACS-pixel, and validate the performance of our model on it. Extensive experimental results on the public dataset FGADR and our EyePACS-pixel demonstrate that compared with the existing detection and segmentation methods, the proposed method can identify lesions accurately and comprehensively, and obtain competitive results using only coarse-grained annotations.

## 1. Introduction

Diabetic retinopathy (DR) is one of the most common complications of diabetes and one of the leading causes of visual impairment in the working-age population. Fortunately, timely diagnosis can prevent further deterioration of the lesions, thus reducing the risk of blindness. During the diagnosis of DR, the ophthalmologist completes the comprehensive diagnosis by identifying the lesion attributes on the fundus image, such as microaneurysm (MA), hemorrhage (HE), exudate (EX), cotton wool spots (CWS), neovascularization (NV), and intraretinal microvascular abnormalities (IRMA). However, due to the difficulty in identifying certain lesions, this process can be time-consuming and labor-intensive. Automatic DR-aided diagnosis methods use deep learning models to extract features from the fundus image to complete the location of the lesions, and the results can be provided to ophthalmologists for further diagnosis. At the same time, with the maturity of automatic DR-assisted diagnosis technology, the requirements for deep learning models in clinical applications are also increasing. For example, it is expected to have the ability to be used across medical institutions. In conclusion, cross-domain localization of DR lesions is becoming a concern of both academia and industry.

**Figure 1:**
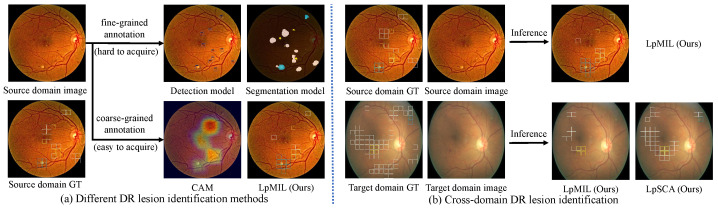
(**a**) Different DR lesion identification methods, including models trained with fine-grained annotations represented by detection models and segmentation models, and models trained with coarse-grained annotations represented by CAM and LpMIL. Using only coarse-grained annotations, our LpMIL not only achieves better lesion identification performance than CAM, but also achieves results that are competitive with detection and segmentation models. (**b**) Directly applying our LpMIL trained on the source domain to the target domain results in severe performance degradation, while our LpSCA improves cross-domain lesion identification performance through the semantic constrained adaptation method. “GT” denotes ground truth.

In recent years, with the development of deep learning, as shown in Figure 1a, several lesion identification models have been proposed to assist ophthalmologists in the diagnosis of DR. Models [1,2,3,4] trained with fine-grained annotations such as pixel-level annotations or bounding box annotations have been proposed and have achieved acceptable results in DR lesion identification. However, the application of these models is limited due to the time-consuming manual annotation. Therefore, some methods [5,6,7] attempt to accomplish both DR grading and lesion identification using only coarse-grained annotations such as grading labels or lesion attribute labels. However, due to the limited supervision provided by coarse-grained annotations, these methods tend to be biased on the most important lesion regions while ignoring trivial lesion information. In addition, in clinical applications, image quality and imaging performance vary due to the different image acquisition equipment used in different healthcare facilities, the direct application of models on other datasets will suffer huge performance losses (Figure 1b), which greatly limits the flexibility and scalability of these deep learning methods.

Motivated by the above observations, we propose a novel cross-domain weakly supervised DR lesion identification method. First, we propose the novel lesion-patch multiple instance learning method (LpMIL), which achieves both image-level supervision and patch-level supervision. Specifically, it utilizes patch-level lesion predictions generated by fully convolutional networks and a specified threshold to generate soft patch-level pseudo-labels, enabling patch-level supervision. At the same time, image-level predictions are obtained by the max-pooling aggregation for image-level supervision. Besides, to fully identify lesions of different sizes, we also introduce a multi-scale fusion method to fuse the features extracted by the backbone. Next, based on LpMIL, we propose a semantic constraint adaptation method (LpSCA) to facilitate the application of the model across medical institutions. A semantic constrained loss is constructed using grading labels, which introduces sufficient medical prior information and improves the performance of cross-domain lesion identification. Finally, since there is no public large-scale fine-grained annotated DR dataset to conduct experiments and verify the effect of our model, we perform secondary annotation on the open-source EyePACS dataset to obtain the largest fine-grained annotation dataset, EyePACS-pixel, and verify the cross-domain identification performance of our model.

The main contribution of our work is as follows:

- We are the first to define DR lesion identification as a multi-label classification task, and propose a novel lesion-patch multiple instance learning method (LpMIL) to achieve it.
- We propose a semantic constraint adaptation method (LpSCA) to improve cross-domain DR lesion identification performance.
- We construct the largest fine-grained annotation dataset EyePACS-pixel, which can provide a data basis for DR lesion identification.
- Extensive experiments conducted on the public datasets FGADR and EyePACS-pixel show that, with only coarse-grained annotations, the proposed method can achieve competitive results compared with the existing dominant detection, segmentation, and weakly supervised object localization methods.

## 2. Related Work

### 2.1. Diabetic Retinopathy Lesion Identification

To complete automatic DR lesion identification, many DR lesion identification methods based on pixel-level or bounding box annotations have been proposed. Yang et al. [8] propose a two-stage Convolutional Neural Network (CNN) for DR grading and lesion detection, which uses the lesion detection results to assign different weights to the image patch to improve the performance of DR grading. Li et al. [9] adopt the object detection model to extract lesion features from fundus images for DR grading. Using a small number of pixel-level annotations, Foo et al. [10] propose a multi-task learning approach to simultaneously complete the tasks of DR grading and lesion segmentation. Zhou et al. [2] propose the FGADR dataset, on which DR lesion segmentation is performed. However, the application of these methods is limited due to the difficulty of obtaining fine-grained annotations. Therefore, researchers attempt to accomplish both DR grading and lesion identification using only coarse-grained grading labels. Wang et al. [5] utilize the attention map to highlight suspicious areas, and complete DR grading and lesion localization at the same time. Sun et al. [6] formulate lesion identification as a weakly supervised lesion localization problem through a transformer decoder, which jointly performs DR grading and lesion detection. Different from previous methods, we define DR lesion identification as a multi-label classification problem for patch-level lesion identification and use a cross-domain approach to enable the model to be used across healthcare institutions.

### 2.2. Multiple Instance Learning

Multiple instance learning has become a widely adopted weakly supervised learning method [11,12,13,14,15,16]. Many works have studied different pooling functions combined with instance embedding or instance prediction to accomplish bag-level prediction [17,18,19,20]. However, the pooling function itself cannot provide sufficient information, and supervision can only be retained at the bag level, which severely limits the effect of instance-level prediction. To address this problem, some approaches introduce artificial instance labels by specifying thresholds to provide both bag-level and instance-level supervision. Zhou et al. [18] use specified thresholds to directly assign positive or negative labels based on prediction scores, providing supervision for all instances. Morfi et al. [21] propose MMM loss for audio event detection. This loss function provides supervision for instances of extreme prediction scores according to the specified threshold and obtains bag level prediction through average pooling aggregation for bag level supervision. Seibold et al. [22] use a more customized way to create soft instance labels, flexibly providing supervision for all instances, and apply it to the pathological localization of chest radiographs pathologies.

To the best of our knowledge, these methods have not been applied to automatic DR detection. In this paper, we use the multiple instance learning method for weakly supervised DR lesion identification.

### 2.3. Domain Adaptation

Domain adaptation is a subtask of transfer learning, which maps features of different domains to the same feature space, and utilizes the labels of the source domain to enhance the training of the target domain. The mainstream approach is to learn the domain invariant representation using adversarial training. DANN [23] pioneers this field by training a domain discriminator to distinguish the source domain from the target domain, and training a feature extractor to cheat the discriminator to align the features of the two domains. CDAN [24] utilizes the discrimination information predicted by the classifier to condition the adversarial model. GVB [25] improves adversarial training by building bridging layers between the generator and the discriminator. MetaAlign [26] treats domain alignment tasks and classification tasks as meta-training and meta-testing tasks in a meta-learning scheme for domain adaptation. However, these methods lack sufficient medical prior information to achieve satisfactory results. Therefore, we propose a semantic constraint adaptation method using the common grading labels of the diabetic retinopathy dataset, and achieve the cross-domain utilization of lesion attribute labels.

## 3. Materials and Methods

### 3.1. Datasets

Currently, although there are some public DR datasets, such as [2,27,28], only FGADR [2], IDRiD [27] contains pixel-level annotations of lesions. Since FGADR is used to train the model, and the amount of data contained in IDRiD is extremely limited, to evaluate the effectiveness of our LpSCA, we construct a fine-grained lesion identification dataset based on EyePACS [28]. Our dataset contains 4401 images with corresponding pixel-level lesion annotations. Due to the requirements of the evaluation experiment, the annotated lesions include HE, CWS, and EX. The number of lesions for each dataset is shown in Table 1.

#### 3.1.1. Our EyePACS-Pixel Dataset

**Figure 2:**
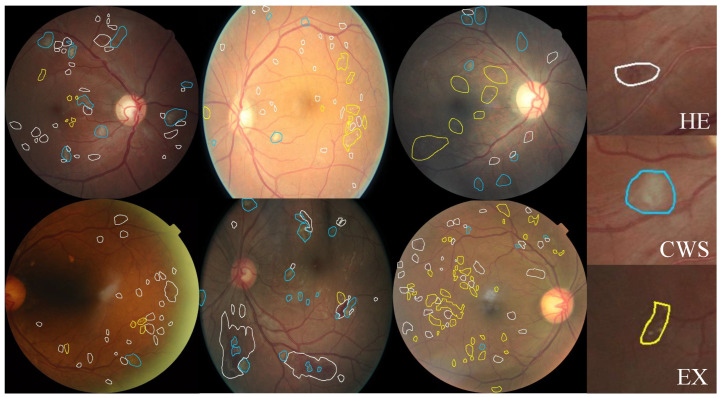
Examples of pixel level annotations from our EyePACS-Pexel dataset. White, blue and yellow indicate HE, CWS, and EX, respectively.

Since our main goal is to build a dataset containing annotated pixel-level DR lesions, we prefer fundus images that contain more lesions. Therefore, we use FGADR to train our LpMIL and apply it to the test set of EyePACS. We select images with lesions predicted by our LpMIL and filter out images with a grade greater than 0 for labeling. Three ophthalmologists (two residents and one attending physician) are invited to annotate the data. Two residents make the preliminary annotation, and the attending physician is responsible for the final verification. This dataset has been approved by the Biological and Medical Ethics Committee of Beihang University (No. BM20230242). Some examples of annotations are shown in Figure 2.

The images in the dataset all contain at least one annotated lesion. The distribution of lesion counts is shown in Table 1. Through observation, we find that HE and EX are two common lesions in DR images, while CWS appeared relatively less frequently.

In our experiment, this dataset is only used to evaluate the cross-domain lesion identification performance of the model.

#### 3.1.2. FGADR Dataset

FGADR dataset [2] contains 1842 fundus images in five DR categories including pixel-level annotations of HE, MA, EX, CWS, NV, and IRMA. Due to the small size of MA and limited training data for IRMA and NV, it is difficult for state-of-the-art semantic segmentation models to achieve satisfactory results on MA, IRMA, and NV. Therefore, excluding MA, IRMA, and NV, we only conduct experimental evaluations for HE, CWS, and EX. We randomly divide it into 1474 training images and 368 testing images, the training set is used for the training of our LpMIL and LpSCA, and the test set is used for the evaluation of lesion identification.

#### 3.1.3. EyePACS Dataset

EyePACS dataset [28] contains 88,702 images in five DR categories, of which 35,126 images are used for training, 10,906 images are used for validation, and 43,670 images are used for testing.

### 3.2. Methods Overview

**Figure 3:**
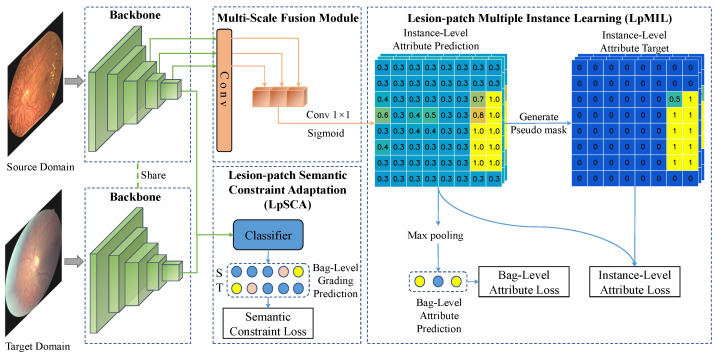
The architecture of our model.

In Figure 3, the images are processed by fully convolutional networks including the backbone and the multi-scale fusion module to obtain patch-level classification predictions for each lesion. The number of patches is related to the size of the feature map, which is determined by the backbone and the input size. Given a set of source domain images $X_{s}$ with lesion attribute labels $Y_{a,s}$ and grading labels $Y_{g,s}$ and a set of target domain images $X_{t}$ with only grading labels $Y_{g,t}$. The purpose of LpMIL and LpSCA is to train the backbone network and the multi-scale fusion module to predict patch-level lesion attribute labels of $X_{s}$ and $X_{t}$, respectively. To achieve this, we first extract the feature $F_{s}$ from the source domain image using the backbone, and then the last few layers of $F_{s}$ are fed into the multi-scale fusion module to fuse the feature maps of different scales, and finally, the LpMIL perform bag-level and instance-level supervision using lesion attribute labels $Y_{a,s}$, where bags and instances correspond to images and patches in the images, respectively. In a cross-medical institution scenario, that is, across different datasets, we use the same backbone to extract the feature $F_{t}$ from the target domain image, and then the LpSCA uses the grading labels $Y_{g,s}$ and $Y_{g,t}$ to perform domain adaptation on the last layer of $F_{s}$ and $F_{t}$ to improve cross-domain lesion identification performance. In the following subsection, we will describe the specific implementation of the above method in detail.

### 3.3. Lesion-Patch Multiple Instance Learning for Lesion Identification

#### 3.3.1. Multi-Scale Fusion Module

Since the lesions in the fundus images are of different sizes, it is difficult to preserve the location information of the lesions after the multi-layer convolution operation. Therefore, we propose a multi-scale fusion module to detect lesions of different sizes. As shown by the multi-scale fusion module in Figure 3, given the feature $F_{s}$ corresponding to the source domain image $X_{s}$, we apply a convolutional layer to convert the outputs of the last few layers of $F_{s}$ into $F_{l,s}$ with the same spatial size, where l∈{1,…,L} and *L* is a hyperparameter that can be manually selected. These features $F_{l,s}$ are transformed into instance-level lesion attribute predictions through convolution and sigmoid operation after concatenation.

#### 3.3.2. Lesion-Patch Multiple Instance Learning

Given lesion attribute prediction $p_{i,j}^{c}$ obtained from the multi-scale fusion module. In multiple instance learning, $p_{i}^{c}=1$ if and only if there is at least one $p_{i,j}^{c}=1$, hence we define

(1) $$p_{i}^{c}=\max_{j}p_{i,j}^{c},$$

where $p_{i,j}^{c}$ is the attribute prediction of the *c*-th class generated by the *j*-th instance of the *i*-th bag from the source domain and $p_{i}^{c}$ is the attribute prediction of the *c*-th class generated by the *i*-th bag from the source domain. Bags and instances correspond to images and patches in the images, respectively.

We refer to [22] and use Self-Guiding Loss (SGL) for multiple instance learning. The specific example is shown in the LpMIL of Figure 3. Unlike the standard method, which only contains bag-level supervision, our method includes bag-level supervision and instance-level supervision. The specific implementation of the two kinds of supervision will be described later.

Like the multi-label classification task, we use a regular loss function L such as the binary cross-entropy loss function to compute the bag-level loss function:

(2) $$L_{\mathrm{Bag}}\left(X_{s},Y_{a,s}\right)=\frac{1}{C\cdot N}\sum_{c}\sum_{i}L\left(p_{i}^{c},y_{i}^{c}\right),$$

where *C* is the number of lesion categories, *N* is the total number of samples, and $y_{i}^{c}$ is the *c*-th lesion attribute of the *i*-th sample from the source domain.

In multiple instance learning, there is an assumption that networks trained just from bag-level annotations will inevitably assign some positive instances a noticeably higher predicted score than most negative instances. Therefore, after initial training, we think that the labels of instances with high predictions should be positive, those with low predictions should be negative, and those predictions close to the decision boundary are ambiguous, and use these labels as instance-level pseudo-labels. The main operations are as follows:

To address the problem of imbalanced data, we perform max-min normalization on the prediction $p_{i,j}^{c}$:

(3) $$\theta_{i,j}^{c}=\frac{p_{i,j}^{c}-\min\left(p_{i,j}^{c}\right)}{\max\left(p_{i,j}^{c}\right)-\min\left(p_{i,j}^{c}\right)}.$$

According to previous assumptions, we define the upper threshold $\delta_{h}$ and the lower threshold $\delta_{l}$ ($\delta_{h}+\delta_{l}=1,\delta_{h}>\delta_{l}>0$), so that labels of instances with predictions greater than the upper threshold $\delta_{h}$ are positive, those below the lower threshold $\delta_{l}$ are negative, and labels of instances close to the decision boundary are normalized predictions to push them towards a certain class, and pseudo-labels are defined as follows:

(4) $$M_{i,j}^{c}=\left\{\begin{array}{cc} 0 & ,\;\mathrm{if}\;\theta_{i,j}^{c}<\delta_{l}\;\mathrm{or}\;y_{i}^{c}=0\\ \theta_{i,j}^{c} & ,\;\mathrm{if}\;\delta_{l}\leq \theta_{i,j}^{c}\leq \delta_{h}\\ 1 & ,\;\mathrm{if}\;\delta_{h}<\theta_{i,j}^{c} \end{array}\right..$$

Next, we use a regular loss function L such as the binary cross-entropy loss function to construct the instance-level loss function:

(5) $$L_{\mathrm{Inst}}\left(X_{s},M\right)=\sum_{i}\sum_{c}\sum_{j}2^{\alpha_{i}^{c}-1}\cdot L\left(p_{i,j}^{c},M_{i,j}^{c}\right).$$

We use the weight $\alpha_{i}^{c}$ to adjust the impact of instance-level loss during training, and $\alpha_{i}^{c}$ is defined as follows:

(6) $$\alpha_{i}^{c}=\max\left(\max_{j}\left(p_{i,j}^{c}\right)-\underset{j}{median}\left(p_{i,j}^{c}\right),1-y_{i}\right).$$

In multiple instance learning, there is an assumption that there are generally fewer positive instances in the positive bag, so the median in the predictions of the well-trained model will be low. For positive bags, if the model can distinguish positive and negative instances well, then we will assign a higher value of $\alpha_{i}^{c}$ to increase the weight of the instance-level loss. Whereas, if the model cannot distinguish positive and negative instances well, then we will assign a lower value of $\alpha_{i}^{c}$ to reduce the weight of instance-level loss. For negative bags, since the instance labels are deterministic, we set $\alpha_{i}^{c}$ to 1 to increase the weight of the instance-level loss. The loss function of our LpMIL is defined as

(7) $$L_{LpMIL}\left(X_{s},Y_{s},M\right)=L_{\mathrm{Bag}}+\lambda \cdot L_{\mathrm{Inst}},$$

where λ represents the weight hyperparameter for instance-level loss.

### 3.4. Lesion-Patch Semantic Constraint Adaptation for Domain Adaptation

Due to the differences in the distribution of different fundus datasets, directly applying a model trained in the source domain to the target domain will result in severe performance degradation. To address this issue, based on LpMIL, we propose a semantic constraint adaptation method (LpSCA), which utilizes grading labels to construct the semantic constrained loss for domain adaptation. Given the corresponding features $F_{s}$ and $F_{t}$ of the source domain image and the target domain image, we use the classifier to obtain the corresponding classification predictions $p_{i,s}$ and $p_{i,t}$, and use the cross-entropy loss function $L_{\mathrm{CE}}$ for supervision:

(8) $$L_{\mathrm{SCA},s}\left(X_{s},Y_{g,s}\right)=\frac{1}{N}\sum_{i}^{N}L_{\mathrm{CE}}\left(p_{i,s},y_{i,s}\right),$$

(9) $$L_{\mathrm{SCA},t}\left(X_{t},Y_{g,t}\right)=\frac{1}{N}\sum_{i}^{N}L_{\mathrm{CE}}\left(p_{i,t},y_{i,t}\right),$$

(10) $$L_{SCA}\left(X_{s},Y_{g,s},X_{t},Y_{g,t}\right)=L_{\mathrm{SCA},s}+L_{\mathrm{SCA},t},$$

where $y_{i,s}$ is the grading label of the *i*-th sample from the source domain, and $y_{i,t}$ is the grading label of the *i*-th sample from the target domain.

The overall loss function of LpSCA is defined as

(11) $$L_{\mathrm{LpSCA}}\left(X_{s},Y_{a,s},Y_{g,s},X_{t},Y_{g,t}\right)=L_{\mathrm{LpMIL}}+L_{\mathrm{SCA}}.$$

## 4. Results

### 4.1. Evaluation Metrics

Since the output of our weakly supervised lesion identification network is a patch-level binary classification result, we transform the lesion identification problem into a multi-label classification problem. Therefore, we use precision, recall, and F1-score as evaluation metrics for lesion identification. Specifically, a 512×512 image can be transformed into a patch-level lesion classification result of 16×16×3 through the processing of our model, where 3 is the number of lesion categories. After determining a threshold, we calculate the precision and recall of each lesion on 16×16 patches and then calculate the F1-score. For simplicity, all the results are the average F1-score under different thresholds T∈{0.5,0.6,0.7,0.8,0.9}.

### 4.2. Implementation Details

In this work, we use ResNet50 [29] as our backbone network to extract features by removing the global average pooling layer and fully connected layers. The fundus image is resized to 512×512 as the input to the network. We set the parameter λ=4 to keep the two losses at similar magnitudes. The parameter *L* of the multi-scale fusion module and the parameter $\delta_{l}$ of LpMIL will be discussed in the next subsection. In particular, the output of the multi-scale fusion module is the patch-level lesion classification result of 16×16×3, which is determined by the number of downsampling times of ResNet50 and the number of lesion categories. With a learning rate of $1\times 10^{-3}$ and a batch size of 128, all our models are trained for 80 epochs using the Adam optimizer and cosine annealing strategy.

### 4.3. Ablation Studies

#### 4.3.1. The Choice of *L* in the Multi-Scale Fusion Module

In this part, we analyze the effect of the hyperparameter *L* in our LpMIL, where *L* is the number of feature layers for multi-scale fusion. The lesion identification performance of our LpMIL on the FGADR dataset with different *L* is shown in Table 2. The results show that the performance of lesion identification improves as *L* increases, and the best results are obtained when L=3. However, as *L* continues to increase, the results instead decrease. We believe that the initial increase in *L* enlarges the receptive field, allowing the model to observe tiny lesions. When L=4, the semantic information of the previous layer is too weak, resulting in a decline in the performance of lesion identification. Therefore, we set *L* to 3 for better performance.

#### 4.3.2. The Choice of the Threshold $\delta_{l}$ in LpMIL

In this part, we only analyze the effect of hyperparameter $\delta_{l}$ in our LpMIL, because $\delta_{l}+\delta_{h}=1$. Table 3 shows the lesion identification results of our LpMIL on the FGADR dataset with different $\delta_{l}$. When $\delta_{l}=0.4$, the lesion identification performance reaches the best. We think that a high threshold enables the model to learn the characteristic information of lesions more smoothly and avoid introducing bias. In the following experiments, we set $\delta_{l}$ to 0.4.

### 4.4. Comparisons with State-of-the-Art Methods

In this section, we compare our model with a series of lesion identification models such as Faster R-CNN [1], U-net [3], CAM [30] and ADL [31]. The first two models are trained with fine-grained annotations, and the latter two are CAM-based weakly supervised object localization methods that use coarse-grained lesion attributes for supervision. For all experiments, we convert their predictions to the same patch-level predictions as our model and then evaluate the results.

#### 4.4.1. Lesion Identification Performance

The lesion identification results on FGADR are shown in Table 4. The performance of two weakly supervised object localization methods, CAM and ADL, is greatly surpassed by our LpMIL. We think that these two methods perform poorly due to the GAP bias of assigning higher weights to smaller activation regions and the instability of using the maximum value of the class activation map as a threshold reference. Compared with Faster R-CNN and U-net, two models trained with fine-grained annotations, LpMIL achieves competitive results and even surpasses these two models in some metrics, which proves the effectiveness of our LpMIL.

**Figure 4:**
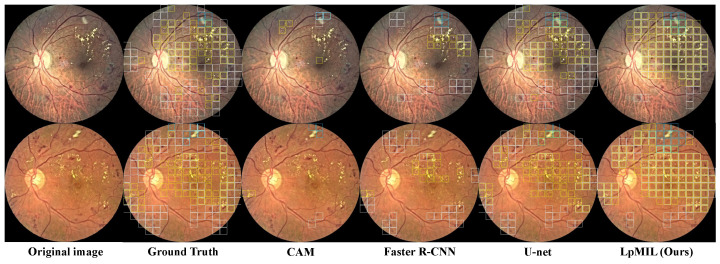
Using the patch-level prediction results, we compare the lesion identification visualization results of LpMIL on the FGADR dataset with other models, where the white, blue, and yellow boxes represent HE, CWS, and EX, respectively.

Figure 4 shows the qualitative results. We can observe that CAM can only identify a small number of lesions, ignoring the majority of lesions. U-net trained with pixel-level annotations can detect lesions in fundus images well, while Faster R-CNN trained with bounding box annotations detects lesions relatively accurate but not comprehensively. Although the identification performance is not as good as U-net, our model can detect most lesions in different regions, which also highlights the superiority of our LpMIL.

#### 4.4.2. Cross-Domain Lesion Identification Performance

In addition to the above baseline trained on FGADR, we also transfer the domain adaptation method of DANN [23] to our LpSCA, replacing the semantic constraint adaptation method with a domain classifier. As shown in Table 5, our LpMIL achieves better results than U-net and Faster R-CNN, demonstrating better generalization of our LpMIL. We can observe that by using adversarial training for domain adaptation, DANN can achieve better results than our LpMIL. Our LpSCA achieves better results than DANN, proving that our semantic constrained adaptation method can provide more prior information and greatly improve the performance of cross-domain lesion identification.

**Figure 5:**
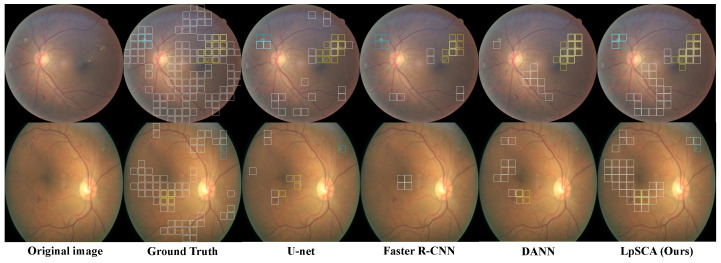
Using the patch-level prediction results, we compare the lesion identification visualization of LpSCA on the EyePACS-pixel dataset with other models, where the white, blue, and yellow boxes represent HE, CWS, and EX, respectively.

Figure 5 shows the qualitative results. We can see that, unlike DANN, our LpSCA can correctly identify CWS, which illustrates that the prior information provided by our semantic constraint adaptation method drives the backbone to learn better lesion features. Compared with U-net and Faster R-CNN, our LpSCA can identify more lesions, which demonstrates the effectiveness of our LpSCA.

## 5. Conclusions

In this paper, we propose a novel cross-domain weakly supervised DR lesion identification method. Specifically, with only coarse-grained annotations, the proposed lesion-patch multiple instance learning method can achieve both image-level and patch-level supervision. The proposed semantic constraint adaptation method leverages the semantic constraints provided by grading labels to improve the cross-domain lesion identification performance of our model. Extensive experiments show that the proposed method can obtain competitive results compared with existing dominant detection, segmentation, and weakly supervised object localization methods. Furthermore, we have noticed that both our model and the compared models have missed a significant number of lesions. Through an analysis of the missed lesions, we believe this can be attributed to certain lesions being of relatively mild severity. These lesions are susceptible to confusion with non-affected areas under different imaging conditions, particularly variations in lighting conditions. We believe that future work could follow a similar approach to how medical professionals review images by conducting a more detailed examination of the surrounding areas where lesions are present.

## Figures and Tables

**Table 1:** A Summary of public DR datasets with fine-grained annotations.

Dataset	Annotation	Images	MA	HE	CWS	EX	IRMA	NV
IDRiD [27]	Pixel-level	81	81	80	40	81	-	-
FGADR [2]	Pixel-level	1842	1424	1456	627	1279	159	49
EyePACS-pixel	Pixel-level	4401	-	4160	1550	2750	-	-

**Table 2:** Lesion identification performance of our LpMIL on the FGADR dataset of different *L*.

*L*	HE	CWS	EX	Mean
1	0.3363	0.2174	0.4529	0.3355
2	0.4086	0.2295	0.5011	0.3797
3	0.4113	**0.2635**	**0.5140**	**0.3963**
4	**0.4261**	0.1891	0.4939	0.3697

**Table 3:** Lesion identification performance of our LpMIL on the FGADR dataset of different $\delta_{l}$.

$\delta_{l}$	HE	CWS	EX	Mean
0.1	0.4092	0.1956	0.4609	0.3553
0.2	**0.4282**	0.2373	0.4664	0.3773
0.3	0.4272	0.2400	0.4824	0.3832
0.4	0.4113	**0.2635**	**0.5140**	**0.3963**

**Table 4:** Performance comparison with state-of-the-art methods on the FGADR dataset. “*” indicates that the model is trained with fine-grained annotations. The best result is bolded and the second best result is underlined.

	HE	CWS	EX	Mean
Faster R-CNN *	0.4029	**0.4329**	0.3002	0.3787
U-net *	**0.5332**	0.3101	**0.5969**	**0.4801**
CAM	0.2123	0.1813	0.3373	0.2437
ADL	0.2192	0.1432	0.3198	0.2274
LpMIL (Ours)	0.4113	0.2635	0.5140	0.3963

**Table 5:** Performance comparison with state-of-the-art methods on the EyePACS-pixel dataset. “*” indicates that the model is trained with fine-grained annotation.

	HE	CWS	EX	Mean
Faster R-CNN *	0.0961	0.2064	0.0818	0.1281
U-net *	0.1659	0.2301	0.3502	0.2487
CAM	0.1851	0.2091	0.3836	0.2593
ADL	0.2126	0.1673	0.3484	0.2428
LpMIL (Ours)	0.2125	0.2632	0.5239	0.3332
DANN	0.2690	0.2783	0.5510	0.3661
LpSCA (Ours)	**0.3985**	**0.3369**	**0.5769**	**0.4374**

## Data Availability

The data presented in this study are available on request from the corresponding author Yunchao Gu.
